# Capacity building in medicines development and regulation in Africa**—**Overview on recent progress

**DOI:** 10.1002/bcp.70770

**Published:** 2026-08-12

**Authors:** Tirhani Maluleke, Emile Malan, Michael Reid, Patrick Bouic, Haylene Nell, Goonaseelan Pillai, Catriona Waitt, Bernd Rosenkranz

**Affiliations:** ^1^ Roche Products Pty Ltd Johannesburg South Africa; ^2^ Fundisa African Academy of Medicines Development Cape Town South Africa; ^3^ Vicore Health Moka Mauritius; ^4^ Gilead Sciences Pty Ltd Johannesburg South Africa; ^5^ Department of Pathology Stellenbosch University Cape Town South Africa; ^6^ Synexa Life Sciences Cape Town South Africa; ^7^ Tiervlei Trial Centre Cape Town South Africa; ^8^ Pharmacometrics Africa NPC Cape Town South Africa; ^9^ Division of Clinical Pharmacology University of Cape Town Cape Town South Africa; ^10^ Department of Pharmacology and Therapeutics University of Liverpool Liverpool UK; ^11^ Infectious Diseases Institute Makerere University College of Health Sciences Kampala Uganda; ^12^ Division of Clinical Pharmacology, Faculty of Medicine and Health Sciences Stellenbosch University Cape Town South Africa; ^13^ Institute for Clinical Pharmacology and Toxicology Charité Universitätsmedizin Berlin Berlin Germany

**Keywords:** Africa, capacity building, health research, healthcare, medicines development, regulation, research policies

## Abstract

**Aims:**

Despite advances in health research and digital innovation, many African countries continue to face substantial resource constraints, fragmented capacity‐building efforts and a shortage of skilled professionals in medicines development and regulatory science. This review explores existing gaps and assesses collaborative approaches to enhancing capacity in this field. It further marks 10 years of sustained contribution by the Fundisa African Academy of Medicines Development towards its advancement.

**Methods:**

A narrative review was conducted to assess capacity‐building initiatives in medicines development and regulation across Africa. Particular focus was placed on the contributions of the Fundisa African Academy of Medicines Development, in collaboration with partners including academia, clinical research sites, industry and regulatory authorities.

**Results:**

Capacity‐building efforts across the continent remain heterogeneous and are often implemented in a fragmented manner. Collaborative initiatives, such as those led by the Fundisa African Academy of Medicines Development and its partners, illustrate the potential to mitigate these gaps through accredited training, targeted skills development and experiential learning programmes. Such initiatives support the strengthening of competencies along the entire medicines development continuum, spanning drug discovery, clinical research and regulatory and health economic evaluation.

**Conclusions:**

Sustainable capacity strengthening in Africa requires coordinated, context‐specific approaches that address the full health research value chain, from investigators to regulatory assessors. Integration of digital health platforms as force multipliers, adoption of standardized competency frameworks such as the WHO Global Competency Framework and development of centralized systems to monitor capacity‐building activities are essential to reduce fragmentation and promote regional collaboration.

**What is known about this subject**

- Resource constraints in many African settings adversely impact healthcare delivery and limit the conduct of locally relevant health research.
- Capacity‐building in medicines development and regulatory science is frequently fragmented, with initiatives delivered in a siloed manner by multiple organizations.

**What this study adds**

- This review delineates key gaps and unmet needs in medicines development and regulation across Africa and outlines pragmatic strategies to address them.
- It highlights the value of coordinated, cross‐institutional collaboration to strengthen workforce capacity and support effective implementation of health research.

## 1 INTRODUCTION

WHO reports substantial progress in the number of African National Regulatory Authorities (NRAs) achieving Maturity Level 3 (ML3) status.^1^

The following countries have reached ML3 status, whereas the remaining countries operate at lower maturity levels: Ghana, Tanzania, Nigeria, South Africa, Egypt (first African country to achieve ML3 for both medicines and vaccines regulation), Zimbabwe, Senegal, Rwanda and Ethiopia.

These developments demonstrate continent‐wide strengthening of regulatory oversight capacity, including product authorization, pharmacovigilance, adverse event monitoring, market surveillance, laboratory oversight and inspection systems.

The efficiency and maturity of regulatory systems directly correlate with healthcare delivery and patient access to medicines. True healthcare access and health gains can only be achieved if sound regulatory systems are in place to ensure medical products are safe and effective.^2^ Historically, the mandatory individual review of products by multiple countries, each with distinct capability challenges, disparate nonscientific requirements and lengthy timelines for manufacturer responses, has led to increased complexity and delayed product approval timelines.^3^

These contributed to marketing authorization of a novel medicine in Sub‐Saharan African countries taking an average of 4–7 years after it was approved in a mature, high‐income market like the United States.^4^ The African countries reaching ML3 can drastically improve healthcare delivery by fostering a culture of trust and regulatory reliance. A failure to leverage or rely on reviews already performed by competent, mature authorities significantly contributes to these delays, hindering timely access to high‐quality, safe and efficacious medical products.^3^ The lack of regulatory system maturity ultimately impedes patients' access to life‐saving medicines.^2^ Less mature agencies should rely on the decisions and scientific assessments of ML3 African agencies or Stringent Regulatory Authorities. However, utilizing facilitated regulatory pathways effectively requires a highly skilled workforce.^5^

Africa imports approximately 94% of its medicinal and pharmaceutical products, exposing its markets to severe vulnerabilities.^6^ A 2018 systematic review revealed that 18.7% of substandard and falsified medicines were found in the African market, representing the highest percentage globally. In the UNODC report, it was noted that fake medicines kill almost 500 000 Sub‐Saharan Africans annually.^6^ Regulatory harmonization and reliance initiatives and the African Medicines Agency (AMA) should leverage the expertise of ML3 agencies to integrate continental post‐marketing surveillance, pharmacovigilance and rapid alert systems. By pooling data on adverse drug reactions and substandard products into a centralized database, authorities can trigger rapid, continent‐wide market withdrawals and combat the proliferation of fake medicines.^4^, ^7^

## 2 GAPS AND NEEDS IN AFRICAN MEDICINES DEVELOPMENT AND REGULATION

The African continent has faced considerable challenges due to resource‐limited settings, which significantly impact the healthcare and societal framework, evidenced by workforce shortages, inadequate health funding (which is mainly provided by international sponsors and donors^8^), fragile health infrastructure and complex socioeconomic factors that contribute to ongoing disease burdens and inequalities.^9^, ^10^ These challenges, poor research policies and facilities as well as lack of research collaborations and/or networks also impede health research on the continent.^8^

Despite continuous progress in developing capacities, advances in innovation and healthcare research, there are not enough highly skilled individuals with diverse experience in medicines development and regulatory oversight.^11^, ^12^, ^13^

The limited number of health research organizations in Africa suggests under‐utilization of the available skills and expertise in the continent and along with the limited and/or lack of appropriate health research systems encourages brain drain.^8^, ^12^ Strengthening capacity development in medicines development and regulatory science in Africa will require sustained collaboration, coordinated monitoring of initiatives and the strategic use of digital platforms to scale training and knowledge exchange. Development of clear career pathways in medicines development and regulation will be essential to maintain talented scientists. This requires proper accreditation of well‐designed training programmes and the establishment of a cooperative network of training institutions.

Examples of gaps identified in the African medicines development and regulation include the following:

- A critical lack of human resource capacity to fully execute regulatory mandates, particularly a shortage of toxicologists and specialized experts required to assess non‐clinical data for new chemical entities (NCEs).^2^ This shortage prolongs assessment timelines for NCEs and impedes patient access.
- In the absence of validated benefit–risk frameworks, clinical assessments have historically relied on subjective ‘gut decisions’ and the personal risk tolerance of reviewers, leading to divergent regulatory outcomes compared to well‐resourced reference agencies.^14^
- Many African NMRAs lack robust information technology (IT) and electronic tracking systems, which hinders application efficiency.^3^, ^15^ In addition, there is a widespread failure to publish public assessment reports (with the exception of Tanzania), which limits regulatory transparency.^2^, ^16^
- Unmet needs in disease burden: Developing nations face an increasing prevalence of non‐communicable diseases, yet agencies are often inadequately resourced to regulate NCEs aimed at these conditions or to deploy facilitated regulatory pathways during health emergencies.^2^

Although significant impact is noted in regulatory assessment of general medicines in African agencies, there is a crucial disparity in biosimilar regulatory knowledge. Recent data show that only 5% to 10% of regulatory clinical assessors enrolling in training programmes have prior exposure to biosimilar assessments.^5^ Addressing this specific skills gap through targeted training is essential to improve patient access to these complex and necessary treatments.

These persistent gaps highlight the need for coordinated and sustainable capacity‐building initiatives that can strengthen expertise in medicines development and regulatory science across the continent. In response to these challenges, several collaborative initiatives have emerged to strengthen training and professional development across Africa.

In recognition of the rapid digital advancements, there is an increasing need to adapt educational approaches and capacity‐building initiatives to a more digitally enabled environment. The US Department of Education highlights the importance of adapting education systems to the growing influence of digital technologies and artificial intelligence in lifelong learning.^17^ One example is the application of data science in health research, which leverages insights from heterogenous data sources to facilitate development of targeted interventions, optimization of resource allocation and implementation of evidence‐based strategies.^9^ Though the activities highlighted by Kiosia et al.^9^ demonstrate a significant impact in capacity development across different areas of health, fragmentation remains a challenge. Coordination, monitoring mechanisms and sustainability plans have not yet been established. Several early‐stage digital health concepts and initiatives are being piloted at local, national, regional and global scales and therefore in future digitization in health is expected to serve as a ‘force multiplier’,^18^ accelerating the convergence and/or synergy of the different interventions to address the silos and fragmentations. With research and development of novel medicines that are safe, effective and of good quality becoming increasingly complex over the past decades, compounded with the use of advancing technologies,^19^ there is an increasing need to accelerate capacity building, skill transfer and knowledge sharing through evidence‐based and/or impactful collaborations across Africa.^20^, ^21^

Capacity building, as defined by the United Nations, is the process of developing and strengthening the skills, instincts, abilities, processes and resources that organizations and communities need to survive, adapt and thrive in a fast‐changing world.^22^ The UN further highlights transformation as an essential ingredient in capacity‐building, which goes beyond performing tasks to changing mindsets and attitudes. Further, capacity building in health research is defined as enhancing the capacity, at the researcher and institution levels, to conduct, manage, disseminate and apply research, while enabling national and sub‐national research systems to effectively support research and the linkages between research, practice, innovation and policy development.^23^

Capacity building in medicines development and regulation in Africa has, over the past decade, progressed from a capability gap to an enabler of growth for the global pharmaceutical industry. Africa's rapidly expanding pharmaceutical markets, increasing clinical trial activity and growing disease complexity were already outpacing the regulatory and scientific workforce capacity 10 years ago.^21^ One such initiative is the Fundisa African Academy of Medicines Development, which aims to promote structured education and collaboration in medicines development and regulation.

Since then, investment in regulatory science and structured training programmes, and institutional strengthening has been reshaping the environment.

The establishment of AMA during this period has signaled continental commitment to regulatory coordination and reliance.

Thus, building the necessary capabilities will help reduce uncertainty in development and registration timelines, enabling earlier inclusion of African countries in global clinical programmes while supporting more predictable pharmaceutical market entry strategies.

Capacity building across the health and research sector should be tailored to provide the output required by the various stakeholders. For regulatory agencies, the development and maintenance of competent and diverse expertise for the assessment and evaluation of scientific data ranging from clinical trials dossiers, marketing authorization applications, pharmacovigilance, licencing and inspections to regulatory monitoring are critical. Upskilling personnel in analysing health information, policymaking and impact assessment is the focus at the government and public sector level. For example, the European Medicines Agency (EMA) supports skills development through various programmes for regulatory staff, including non‐EU regulators (https://arlo.pmxafrica.org/resource/recu3ajxirx9uigia). The competency framework for regulators of medicines established by WHO^24^ is aimed at harmonizing workforce development efforts for the regulation of medicines by establishing internationally accepted sets of organizational and role‐specific competencies.

Capacity‐building of healthcare professionals entail gaining an appreciation of the value of research and keeping up to date with contemporary research, applying findings in clinical decision‐making processes, to knowledge creation and conducting clinical research that has the potential to enhance the quality of patient care and, ultimately, embed discovered knowledge into practice.^25^ For health research and development, this includes advanced laboratory specializations, specialized and multidisciplinary research and support teams across the value chain, as well as skills in the implementation of health services.^8^

More recently, capacity building has been expanded to patients and consumers (https://www.ema.europa.eu/en/partners-networks/patients-consumers/training-resources-patients-consumers). Skills include contribution to scientific advice procedures, scientific‐advisory‐group meetings and reviewing medicines' information for patients.

For capacity development to be successful, joint efforts from all relevant stakeholders are required to ensure sufficient and optimal resources and adequate support and infrastructure.

For multinational pharmaceutical companies, success in capacity building translates into access to diverse patient populations, more representative evidence generation and deeper alignment with global expectations around equity, inclusion and sustainability.

A central repository or database such as the African Regional Learning Observatory (ARLO, https://arlo.pmxafrica.org/), to register the different capacity building activities in the health sector across Africa (at a regional and country level), can showcase opportunities as well as projects with similar objectives thereby mitigating duplication of scarce resources. Fragmentation contributes to the resource constraint settings in Africa and undermines the outcomes and impact of these projects due to their isolated/fragmented nature and diminishes their visibility.

Against this background, several initiatives have emerged to address capacity gaps. One notable example is the Fundisa African Academy of Medicines Development and its partners, whose contributions to training and collaboration across Africa are discussed below which marks 10 years of its sustained contribution towards its advancement.

## 3 A PRACTICAL EXAMPLE: FUNDISA AFRICAN ACADEMY OF MEDICINES DEVELOPMENT (FUNDISA)

In 2014, Fundisa African Academy of Medicines Development was established in Cape Town to provide and promote education and training in medicines development and regulation in South Africa and beyond. Its collaborative approach to capacity building is realized through the engagement of relevant stakeholders from academia, industry, regulatory authorities and other institutions with expertise in their respective fields of practice from various African regions, while also drawing support from the global community as needed.

Fundisa African Academy of Medicines Development in partnership with other organizations has initiated several programmes based on assessments of the local context and gaps identified. In recognition of its more than 10 years of existence in the capacity development sector, an overview of the initiatives and projects that Fundisa African Academy of Medicines Development has initiated or contributed to is summarized in Table 1. Fundisa African Academy of Medicines Development is committed to working closely with its cooperation partners, including academic institutions (Stellenbosch University, University of the Witwatersrand, Charité Universitätsmedizin Berlin, Makerere University and University of Pretoria), government (South African National Department of Health, national regulatory agencies including the South African Health Products Regulatory Authority [SAHPRA], Zimbabwe MCZA, Ghana FDA and European CHMP) and other stakeholders (Pharmacometrics Africa, Tiervlei Trial Centre, PharmaTrain, CP+ Associates, the Gates Foundation, WHO, the International Federation of Associations of Pharmaceutical Physicians and Pharmaceutical Medicine [IFAPP]).

**TABLE 1:** Capacity building initiatives by Fundisa African Academy of Medicines Development together with its partner organizations (details provided at https://www.fundisa‐academy.com/).

Date	Initiative	Partners/collaborators	Key learnings and outcomes
2013^a^–2015	3 Regulatory workshops	Joint initiative with the South African Medicines Control Council (MCC, now South African Health Products Regulatory Authority, SAHPRA) https://www.sahpra.org.za and European Medicines Agency (EMA, CHMP) (https://www.ema.europa.eu)	Medicines regulation in Europe, accelerated approval procedures, anti‐infectives, antimicrobial resistance, vaccines, advanced therapeutic medicinal products (ATMPs), pharmacovigilance, paediatric drug development, generics and biosimilars, medical devices, diagnostics, benefit–risk assessment and capacity building
2013^a^	Medical writing workshop	South African Clinical Research Association (SACRA)	Clinical trial protocols, subject information and informed consent, clinical study reports
2014	Satellite Workshop of the World Congress of Basic and Clinical Pharmacology (WCP2014) ‘Training of medicines development and regulation in emerging countries’^20^	The International Federation of Associations of Pharmaceutical Physicians and Pharmaceutical Medicine (IFAPP) PharmaTrain SAHPRA Hungarian Society for Experimental and Clinical Pharmacology The Bill & Melinda Gates Foundation	Strategies for competency strengthening in medicines development and regulation in low‐and‐middle income countries (LMICs)
Ongoing since 2015	Annual Clinical Investigator (and Site Staff) Certification (CLIC) course (interrupted during COVID‐19 pandemic, 2020–2022) according to PharmaTrain syllabus^26^	Tiervlei Trial Centre, Cape Town, South Africa Pharma Train	Roles and responsibilities of investigators and site staff in accordance with international standards Implementation of novel developments such as digitization of clinical trials, according to globally recognized standards (ICH, WHO, Declaration of Helsinki) Continuous Professional Development (CPD)
2015	Support of Novartis training programme	Novartis South Africa	Evidence‐based medicine, presentation skills, clinical development and research publishing
2016–2021	3 Pharmacoeconomics Workshops (2021 Workshop as hybrid event in Potsdam, Germany and Pretoria, South Africa and virtual participation)	South African National Department of Health PharmaTrain University of Pretoria Faculty of Health Sciences Brandenburg, Germany Potsdam University, Germany Pharmacometrics Africa, Cape Town, South Africa Scenarium, Berlin, Germany	Integration of Health Technology Assessment (HTA) into Universal Health Coverage (UHC) and National Health Insurance (NHI): the recognized need to use HTA to inform resource allocation, evaluate cost‐effectiveness and establish an essential package of care for the NHI, which includes a strategic plan to develop an independent HTA agency in South Africa Evidence‐based decision‐making for clinical guidelines: The conference highlighted the importance of using pharmacoeconomics and HTA to guide the Essential Medicines List (EML) and Standard Treatment Guidelines, ensuring that funded medicines and health technologies provide the best clinical effectiveness, safety, and overall value for money Ethical governance and ‘accountability for reasonableness’: the necessity of applying a robust ethical framework to HTA processes, which requires transparent, inclusive, and independent decision‐making that considers social values, equity, and the financial impact of funding or disinvesting in specific treatments
2019	Novel clinical trial methodologies workshop	Pharmacometrics Africa, Cape Town, South Africa	Modelling and simulations, innovative trial designs, and real‐world evidence
September 2020	Webinar ‘Controlled human infection challenge studies: lessons from Malaria towards COVID‐19’	Pharmacometrics Africa, Cape Town, South Africa CP+ Associates, Basel, Switzerland The COVID‐19 Clinical Research Coalition	Ethical and scientific principles for such studies, risk mitigation steps, applications and practical concerns in the context of COVID‐19 vaccine development
Ongoing since 2021	Annual regulatory assessors course: a 14‐week university‐certified course for African clinical assessors, which actively trains regulatory scientists to conduct abridged assessments, evaluate the acceptability of foreign clinical data, and apply evidence‐based regulatory decision‐making ^5^, ^12^	SAHPRA (South Africa) Medicines Control Authority of Zimbabwe (MCAZ) Ghana FDA Pharmacometrics Africa, Cape Town, South Africa University of the Witwatersrand (Wits), Johannesburg, South Africa German Development Cooperation (GIZ) The Bill and Melinda Gates Foundation	Hands‐on dossier assessments. 197 regulatory scientists from 11 NRAs, and a smaller number from the private sector and academia across Africa enrolled in the programme. Enhanced Knowledge of Regulatory Mechanisms: Between 72% and 90% of participants reported that the training was highly useful. A key learning outcome was a better understanding of regulatory and reliance mechanisms within regional contexts. Establishment of a Sustainable Training Model: To ensure long‐term capacity development, a Train‐the‐Trainer approach was successfully implemented in 2024. By integrating course alumni as faculty members, the programme expanded its mentorship opportunities and ensured that regulatory expertise remains sustained within the African community Post‐Course Mentorship: Whereas the course provided a strong foundation, structured institutional support and formal mentorship are essential for real‐world application and long‐term competency retention Evolution to Flexible Learning Formats: pairing of in‐house alumni mentors to reinforce learning and accommodate professional obligations
2021	Webinar ‘COVID‐19 in Sub‐Saharan Africa’	International Federation of Associations of Pharmaceutical Physicians and Pharmaceutical Medicine (IFAPP)	Management of the Covid‐19 pandemic in Africa, practical experiences, vaccine policies and legal aspects
2022	3rd World Conference on Pharmacometrics (WCoP2022), Cape Town (Organising Committee membership, Finances) (https://wcop2022.org/)	University of Cape Town, South Africa Pharmacometrics Africa, Cape Town, South Africa	Advancing global health and policy through pharmacometrics, highlighting the use of simulations for health equity (particularly noted during the COVID‐19 pandemic) and the development of translational tools to improve combination regimens for tuberculosis Utilizing open‐source and computational modelling tools, with a strong focus on applying in silico models to anti‐infective drug development and practical workflows using platforms like the OSPSuite, R (nlmixr, ggPMX), and NONMEM Improving clinical trial design by equipping attendees with tools for adequately powered, model‐based analysis and fostering direct engagement with clinical pharmacometricians to apply these concepts in real‐world settings
2022	2 Side Meeting sessions at the 3rd World Conference on Pharmacometrics (WCoP2022), Cape Town (https://www.sahpra.org.za/event/3rd‐world‐conference‐on‐pharmacometrics‐wcop/)	SAHPRA CP + Associates, GmbH, Basel, Switzerland Pharmacometrics Africa	Establishment of harmonized, structured training frameworks: Initiatives like the Regional Centres of Regulatory Excellence (RCOREs) successfully developed around 25 curricula and trained over 360 pharmaceutical personnel across Africa. To further harmonize training, a three‐tiered ‘Body of Knowledge’ (Foundation, Specialization, and Advanced) in implementation to standardize learning and align regional programmes with the WHO Global Competency Framework. Enhanced capacity for complex clinical assessments: Intensive Clinical Assessor courses significantly improved regulators' ability to review NCEs, biosimilars, and clinical study data. This directly led to practical outcomes and the successful application of reliance models. Significant expansion of PMX and modelling expertise: programmes like PMX Africa trained nearly 500 scientists across the continent, launching localized initiatives like the Uganda Chapter to deepen foundational knowledge in pharmacokinetics and pharmacodynamics (PK/PD).
2023	Cross‐Disciplinary Mathematical Modelling Symposium^27^	Pharmacometrics Africa Stellenbosch University (School for Data Sciences and Computational Thinking and Division of Clinical Pharmacology), South Africa	Presentations by scientists from different disciplines about concepts and applications of mathematical modelling and simulation: Clinical pharmacology acts as a crucial nexus: Clinical pharmacology identified as the ideal field to seamlessly integrate various scientific disciplines, enabling a holistic approach to complex, interconnected healthcare and environmental challenges Cross‐disciplinary collaboration is essential: There is a critical need to break out of scientific silos, share standardized data, and adapt modelling techniques across fields to reduce duplicated efforts and create more robust, comprehensive models Current limitations must be addressed: Significant challenges remain, including inadequate computing resources for processing big data, major data gaps in low‐ and middle‐income regions like Africa, and the need to effectively communicate complex modelling outcomes to policy‐ and decision‐makers.
2024	Digital Health Africa 2024 Conference^28^	Pharmacometrics Africa Stellenbosch University (School for Data Sciences and Computational Thinking and Division of Clinical Pharmacology), South Africa	Overcoming foundational barriers: Addressing infrastructural limitations, such as unreliable electricity and internet connectivity, and establishing clear regulatory and data governance frameworks are critical for the successful adoption of digital health and AI technologies Developing inclusive, context‐specific solutions: Digital health interventions must be patient‐centred, equitable, and culturally relevant to African communities, which requires active end‐user involvement in their design alongside strong public‐private partnerships Strategic integration and capacity building: Future progress depends on integrating digital health tools into national health policies, conducting rigorous implementation research, and significantly enhancing digital literacy and ongoing training for healthcare professionals
2025	Digital Health Africa 2025 Conference Hybrid multi‐site event (Pretoria, South Africa, Kampala, Uganda, Berlin, Germany) and virtual participation^29^	Charité Center for Global Health, Charité Universitätsmedizin Berlin, Germany The Infectious Diseases Institute, Makerere University College of Health Sciences, Kampala, Uganda Pharmacometrics Africa, Cape Town (South Africa) The Innovation Hub, Pretoria, South Africa	Successful scaling of digital health tools requires a robust systemic foundation built on three core pillars: technical capacity (infrastructure), legal legitimacy (regulation), and effective management (governance) Scientific advancement in areas like artificial intelligence and pharmacometrics is insufficient on its own; a major priority is the safe and practical transition of these complex models from the laboratory to the clinical bedside Sustainable integration of digital health interventions in resource‐constrained environments demands cross‐continental collaborations, extensive capacity building, and long‐term commitments to infrastructure and health policy development

^a^ 2013 Regulatory Workshop and Medical Writing Workshop conducted under the provisional name of ‘Southern African Academy of Medicines Development’.

The success of these conferences, workshops, training courses and symposia demonstrates the impact of collaborations with key stakeholders on the progress of capacity development as well as overall shaping of the health ecosystem in the continent. Effective accredited training programmes are essential for career development in the specialized medicines development and regulation sector.^30^

Although these initiatives continue to develop capacities, validated scientific tools are needed to adequately measure the effectiveness and sustainability of the results attained during these sessions, to track the progress of the collaborations established and enable continued networking opportunities. These should be based on internationally agreed professional frameworks, such as the WHO Global Competency Framework for Regulators (GCF) alongside the WHO Global Benchmarking Tool^24^ for regulatory assessors training, needs identification, implementation and to measure progress.^31^ The WHO framework with adaptation to local needs^12^ can be used as a blueprint for other areas of capacity building in the health and medical research sectors.

The WHO's Global Benchmarking (GBT) provides a systematic scale from 1 to 4 to evaluate the maturity of regulatory systems, helping agencies establish targeted institutional development plans.^2^, ^32^ It facilitates regulatory reliance, harmonization and a benchmarking culture where less mature NMRAs can study and learn from more mature regional counterparts, thereby encouraging continued investment in regulatory system strengthening.^2^ This framework is now being used by the African agencies to shift from fragmented operations to structured, competency‐based workforce development:

- Structured institutional development: The GBT allows agencies to conduct self‐benchmarking to identify critical gaps and establish targeted institutional development plans. By tracking and addressing these gaps, agencies have shown verifiable progress. For example, one NMRA (Pharmacy Board of Sierra Leone) moved from ML1 in 2016 to higher levels by 2021 by explicitly improving its Quality Management Systems (QMS) and pharmacovigilance.^4^, ^33^
- Attaining ML3 Status: Through these systematic assessments, several African agencies (e.g., Tanzania, Ghana, Egypt, Nigeria, South Africa, Zimbabwe, Rwanda and Senegal) have demonstrated stable, well‐functioning and integrated regulatory systems and achieved ML3 status for medicines and/or vaccines.^2^, ^6^, ^7^
- Spillover effect: The transparency and confidence generated by the WHO GBT creates a positive regional momentum. As more leading agencies successfully benchmark their systems and achieve ML3, neighboring countries are directly encouraged to invest in their own regulatory systems, driving a continent‐wide uplift in regulatory maturity.^2^
- Targeted workforce development: The successful attainment of ML3 in countries such as South Africa, Ghana, Tanzania and Nigeria has been supported by enrolling their regulatory scientists in specialized, WHO‐GCF‐aligned programmes.^5^ By training assessors from 11 African countries in the critical review of clinical efficacy, safety data and objective decision‐making, African NRAs are building the reliable, integrated workforce necessary to not only achieve but sustain ML3 status. Through the Annual Regulatory Assessors Course and the facilitated regulatory pathways, SAHPRA has reduced its backlog approval timelines by 68% for both new chemical entities and generics by employing reliance pathways such as verification and abridged review models.^34^ Strategic distribution of workloads across regions by allowing ML3 agencies to take the lead as rapporteurs in joint assessments, whereas less mature agencies serve as reliance partners, thereby conserving limited resources while simultaneously building capacity.^4^, ^7^
- Enhance institutional mentorship: Even when assessors receive advanced training, heavy workloads and limited institutional support can prevent them from applying their new skills independently to speed up reviews.^5^ Integrating ‘train‐the‐trainer’ models and providing post‐course mentorship within NRAs, as successfully implemented in the Annual Regulatory Assessors Course, are mandatory to bridge the gap between learning and real‐world application, driving faster regulatory excellence.

Pharmacometrics Africa, a long‐term partner of Fundisa African Academy of Medicines Development, employs a distinctive capacity‐building approach that yields measurable outcomes. Established as a platform to develop and deliver open‐access educational programmes in quantitative clinical pharmacology in collaboration with local research organizations and academic institutions, it has successfully expanded its African presence by local chapters including Kenya, Nigeria, South Africa, Uganda, Tunisia and Egypt. These chapters apply multiple tactics to provide experiential learning opportunities covering fundamental pharmacometrics concepts, modelling techniques and their application to real‐world problems. Mid‐career scientists have engaged in real‐world data analytics projects, contributing to the development of a skilled workforce in the field. Numerous programme alumni are now actively involved in pharmacometrics units within African universities, consulting groups and local research centres, thereby supporting capacity retention and strengthening research and healthcare infrastructure within their home countries (pmx.africa.org). Systematic documentation of capacity‐building activities across the continent is needed to address the capacity challenges. And equally important is sharing with the relevant stakeholders to mitigate the fragmentation and serve as evidence for others to learn from and reach out to collaborate.

The Clinical Trials Community (CTC) Africa (https://www.ctc.africa), an online portal created to increase the visibility of African clinical trialists and sites in Africa, has been established to provide easy access to African clinical trialists, site feasibility data and regulatory and ethics information all on one platform. Through the use of this platform, there is reduction of unnecessary duplication by researchers and sponsors by providing access to current site feasibility data, to improve access to regulatory and ethics requirements across countries with an online platform for sponsors as well as highlighting gaps and promoting synergy across strategic funders (https://www.ctc.africa).

Collectively, these initiatives illustrate several key factors for capacity development in Africa, including multi‐stakeholder collaboration, accredited training frameworks, experiential learning and alignment with international competency frameworks. However, sustainable impact requires coordinated monitoring mechanisms and stronger integration between digital platforms and training programmes.

## 4 CONCLUSION

Strengthening capacity development in medicines development and regulatory science in Africa will require sustained collaboration, coordinated monitoring of initiatives and the strategic use of digital platforms to scale training and knowledge exchange.

Non‐profit organizations such as Fundisa African Academy of Medicines Development and Pharmacometrics Africa play an important role in strengthening medicines development and regulation in Africa through collaborations with academic institutions, regulatory authorities, industry and global partners. These initiatives demonstrate the value of structured training programmes, cross‐sector collaboration and experiential learning opportunities in developing the next generation of professionals in health research and regulatory science.

Looking ahead, further progress will depend on strengthening coordination and sustainability of capacity‐building efforts across the continent. Establishing mechanisms to systematically document and monitor training activities—such as centralized repositories or regional observatories—could improve visibility, facilitate collaboration and reduce duplication of efforts. In addition, greater integration of digital platforms and data‐driven approaches can expand access to training, support knowledge exchange and enhance regulatory science capabilities.

Continued collaboration among academia, industry, regulatory authorities, governments and international funding partners will be essential to sustain these efforts. By aligning capacity‐building initiatives with global competency frameworks and fostering coordinated regional partnerships, Africa can continue to strengthen its health research ecosystem and support more effective medicines development and regulatory oversight.

## AUTHOR CONTRIBUTIONS

*Manuscript writing—original draft*: Tirhani Maluleke. *Manuscript writing—review and editing*: Emile Malan, Michael Reid, Patrick Bouic, Haylene Nell, Goonaseelan (Colin) Pillai, Catriona Waitt, Bernd Rosenkranz and Tirhani Maluleke.

## CONFLICT OF INTEREST STATEMENT

The authors declare that the research was conducted in the absence of any commercial or financial relationships that could be construed as a potential conflict of interest. Tirhani Maluleke, Emile Malan, Patrick Bouic, Haylene Nell, Goonaseelan (Colin) Pillai and Bernd Rosenkranz are members of the board of directors of the Fundisa African Academy of Medicines Development.

## Data Availability

Data sharing is not applicable to this article, as no datasets were generated or analysed during the current study.
